# Platelet hemostasis in patients with metabolic syndrome and type 2 diabetes mellitus: cGMP- and NO-dependent mechanisms in the insulin-mediated platelet aggregation

**DOI:** 10.3389/fphys.2014.00501

**Published:** 2015-01-05

**Authors:** Tatiana E. Suslova, Alexei V. Sitozhevskii, Oksana N. Ogurkova, Elena S. Kravchenko, Irina V. Kologrivova, Yana Anfinogenova, Rostislav S. Karpov

**Affiliations:** ^1^Federal State Budgetary Scientific Institution “Research Institute for Cardiology,”Tomsk, Russia; ^2^Center of High Technology in the Medicine, Laboratory for Translational Cellular and Molecular Biomedicine, National Research Tomsk State UniversityTomsk, Russia; ^3^Institute of Physics and Technology, National Research Tomsk Polytechnic UniversityTomsk, Russia

**Keywords:** platelets, metabolic syndrome, type 2 diabetes mellitus, nitric oxide, nitric oxide synthase, cyclic guanosine monophosphate

## Abstract

Patients with metabolic syndrome (MetS) and type 2 diabetes mellitus (T2DM) have high risk of microcirculation complications and microangiopathies. An increase in thrombogenic risk is associated with platelet hyperaggregation, hypercoagulation, and hyperfibrinolysis. Factors leading to platelet activation in MetS and T2DM comprise insulin resistance, hyperglycemia, non-enzymatic glycosylation, oxidative stress, and inflammation. This review discusses the role of nitric oxide (NO) in the regulation of platelet adhesion and aggregation processes. NO is synthesized both in endotheliocytes, smooth muscle cells, macrophages, and platelets. Modification of platelet NO-synthase (NOS) activity in MetS patients can play a central role in the manifestation of platelet hyperactivation. Metabolic changes, accompanying T2DM, can lead to an abnormal NOS expression and activity in platelets. Hyperhomocysteinemia, often accompanying T2DM, is a risk factor for cardiovascular accidents. Homocysteine can reduce NO production by platelets. This review provides data on the insulin effects in platelets. Decrease in a number and sensitivity of the insulin receptors on platelets in T2DM can cause platelet hyperactivation. Various intracellular mechanisms of anti-aggregating insulin effects are discussed. Anti-aggregating effects of insulin are mediated by a NO-induced elevation of cGMP and upregulation of cAMP- and cGMP-dependent pathways. The review presents data suggesting an ability of platelets to synthesize humoral factors stimulating thrombogenesis and inflammation. Proinflammatory cytokines are considered as markers of T2DM and cardiovascular complications and are involved in the development of dyslipidemia and insulin resistance. The article provides an evaluation of NO-mediated signaling pathway in the effects of cytokines on platelet aggregation. The effects of the proinflammatory cytokines on functional activity of platelets are demonstrated.

Metabolic syndrome (MetS) and type 2 diabetes mellitus (T2DM) are the factors of cardiovascular risk. MetS comprises an array of pathogenetically interrelated metabolic and clinical abnormalities (insulin resistance, arterial hypertension, and dyslipidemia) and increases risk of atherosclerotic damage of blood vessels. An increase in thrombogenic risk in patients with MetS and T2DM is considered to be caused by platelet hyperaggregation and hypercoagulation due to elevated level of fibrinogen, increased activity of blood-coagulation factors, and hyperfibrinolysis (Medvedev et al., 2004; Matsakaria et al., 2006; Idrisova et al., 2007). Metabolic abnormalities, associated with insulin resistance syndrome, significantly affect functional activity of platelets (Schaeffer et al., 1999; Suslova et al., 2007). Activation of platelets can play an important role in progression of heart failure due to a formation of the microthrombi in the myocardial microcirculation (Serebruany et al., 2002).

According to the results presented by G. G. Petrik and S. A. Pavlishchuk, the augmentation of platelet aggregation activity in T2DM is caused by the altered metabolism: phase I of platelet aggregation is associated with the changes in blood plasma protein and lipid contents; phase II is associated with an increased number of polymorphonuclear granulocytes and dyslipidemia (Petrik and Pavlishchuk, 2008). The study also demonstrated an increase in the mean platelet diameter in T2DM patients (Pavlishchuk et al., 2007). According to other study, the platelet size and cell volume do not differ between T2DM patients and healthy volunteers (Schaeffer et al., 1999).

Several publications provide evidence of putative interrelations between the MetS components and blood platelet count. Kotani K. et al. showed that blood platelet number in the individuals presented with three and more MetS components is significantly higher than in the presence of only one or two components or without MetS components at all (Kotani et al., 2007). Strong positive correlation between blood platelet count and the number of present MetS components was documented upon adjustment for a variety of biosocial factors (age, smoking, alcohol use, and physical activity). Therefore, thrombocytosis can partially explain the augmented cardiovascular risk in MetS patients (Muratova, 2004; Berkovskaya and Butrova, 2009).

There is evidence (Idrisova et al., 2007) that spontaneous platelet aggregation, estimated based on the sizes of the formed aggregates, was significantly higher in MetS patients compared with healthy volunteers. The curves of the mean aggregate sizes and light transmission characteristics suggested that the rates of collagen-induced aggregation of isolated platelets in MetS patients significantly exceeded the corresponding values in group of healthy volunteers (Idrisova et al., 2007). Several publications suggest that platelet sensitivity in T2DM patients increases due to action of the various platelet aggregation inductors including ADP, thrombin, and collagen (Kutti et al., 1986; Bode-Böger et al., 1998; Tokuda et al., 2012). Correlation analysis reveals the presence of direct relationship between the glycated hemoglobin levels and the rates of collagen- and ADP-induced platelet aggregation activities (Rabini et al., 1998; Gruzdeva et al., 2008; Tokuda et al., 2012). The very elevated glycosylation of the platelet membrane proteins, rather than molar ratio of cholesterol and phospholipids, can decrease platelet membrane fluidity in diabetic patients (Muratova, 2004; Vinik and Erbas, 2013). Increased glycosylation of platelet proteins modulates cellular functions in diabetes. In particular, possible glycosylation of calmodulin that modulates activity of nitric oxide synthase (NOS) results in the decreased synthesis of nitric oxide (NO) (Kutti et al., 1986).

The experimental studies demonstrated that C-peptide *in vitro* modulates thrombogenesis (Lindenblatt et al., 2006). In our study, we demonstrated positive association of the basal level of C-peptide with the degrees and rates of the collagen- and ADP-induced platelet aggregation (Suslova et al., 2007; Gruzdeva et al., 2008). These positive correlations suggest interplay between the elevated basal C-protein blood level and platelet aggregation in T2DM. Apparently, insulin resistance, hyperglycemia, and elevated non-enzymatic protein glycosylation in T2DM are the factors causing increased platelet sensitivity to the inductors of platelet aggregation (Gruzdeva et al., 2008).

Some data suggest that hyperglycemia contributes to platelet aggregation whereas normalization of glucose concentration attenuates this process (Dandona and Aljada, 2004). The study by P. Gresele et al. shows that single short-time elevation of the glucose concentration triggers rapid increase in the platelet activation in T2DM patients. This result is confirmed by the increased interaction of platelets with collagen, elevated expression of activation-associated platelet antigen, and augmented urinary excretion of 1-dehydro-TxB2, the marker of platelet activation (Gresele et al., 2010). High glucose concentrations hypothetically activate endothelial NOS in platelets via the osmotic mechanisms involving protein kinase C-β isoform and intracellular calcium increase (Gkaliagkousi et al., 2007).

The molecule of NO is a universal regulator in the cardiovascular, immune, and nervous systems of the organism. NO is synthesized both in endothelial, neural, smooth muscle cells, and in platelets. This molecule thereby mediates autoregulation of platelet activity. NO is a neutral radical with unpaired electron. This molecule has the highest diffusion coefficient compared with other molecules (O_2_ and CO_2_) in the organism and freely penetrates cellular membranes (Malakhov et al., 2009). The synthesis of NO occurs via NO-synthase (NOS) that exists in three isoforms: neuronal (nNOS, NOS-1), inducible (iNOS, NOS-2), and endothelial (eNOS, NOS-3) (Randriamboavonjy and Fleming, 2005). Enzymes catalyze the five-electron oxidation of L-arginine to L-citrulline and NO. Both constitutive and inducible NOSs are expressed in many cell types. Moreover, the isoforms of constitutive NOSs are considered to be parts of two signaling pathways in the cell (Malakhov et al., 2009). There are many publications that reveal the involvement of iNOS in physiological synthesis of NO and impact of eNOS and nNOS on extra synthesis of NO in infection, allergic, and autoimmune diseases (Wahl et al., 2003; Jarazo-Dietrich et al., 2012; Singer et al., 2013). Activation of iNOS is a component of many protective-adaptive reactions (Das and Das, 2008; Ye et al., 2010; Abu-Amara et al., 2012). The basal iNOS-catalyzed NO production and its role in the regulation of vascular tone remain under discussion (Granik et al., 1997; Bondarenko et al., 2002). When eNOS is expressed in plasma membrane of the endotheliocytes (EC) and colocalized with caveolin, its activity is very low. A variety of receptor-dependent stimuli (acetylcholine, bradykinin, histamine, and thrombin) trigger the displacement (extrusion) of eNOS from the complex of caveolin-eNOS and elevate calcium concentration in EC leading to the release of eNOS from caveolin-eNOS complex, its activation by calcium-calmodulin, L-arginine oxidation, and synthesis of small (picomolar) quantities of NO (Oemar et al., 1998; Malakhov et al., 2009). Free fatty acids that are often elevated in obesity inhibit eNOS *in vitro* (Scherrer and Sartori, 1997). According to some studies, platelet eNOS activity is lower in diabetic patients compared with healthy individuals (Randriamboavonjy and Fleming, 2005). Available literature highlights the fact that platelet eNOS can be activated by β2-adrenoceptors that activate adenylate cyclase. Therefore, eNOS activation in platelets is possible due to cAMP increase and protein kinase A (PKA) upregulation which demonstrates close association between cAMP and cGMP systems (Gkaliagkousi et al., 2007).

Activation of iNOS results in synthesis of high NO concentrations enough to stimulate T-cell-mediated immunity and exert cytotoxic effects. iNOS is identified in macrophages, neutrophils, keratinocytes, fibroblasts, chondrocytes, osteoklasts, neurons, astrocytes, various epithelial cells (respiratory, retinal, pigment, renal, tubular, and adenocarcinomatous), hepatocytes, pancreatic β-cells, endotheliocytes, endocardiocytes, and vascular smooth muscle cells. The enzyme is activated by cytokines or bacterial antigens in inflammation as well as by ultraviolet, ozone, nicotinic acid, and hormones affecting cAMP synthesis (adrenalin, glucagon). This NOS isoform generates manifold NO amount compared with other forms of NOSs and does not require Ca^2+^ for this process (Malakhov et al., 2009). Our study demonstrated an increased basal NO production by monocytes isolated from MetS patients perhaps due to the iNOS activation (Suslova et al., 2005).

NO stimulates synthesis of endothelial growth factor, but suppresses smooth muscle cells proliferation and migration thus preventing neointima formation and vascular hypertrophy. Low concentration of this molecule attenuate whereas high concentration stimulate apoptosis and suppress extracellular matrix synthesis thus maintaining normal structure of the vascular wall (Malakhov et al., 2009). NO exerts anti-inflammatory and antithrombogenic properties: (i) suppressing transcription of the anti-inflammatory transcription factor, nuclear factor-kappa-B (NF-kB) (Smolenski, 2012); (ii) inhibiting cytokine-stimulated expression of the adhesive endothelial molecules [VCAM-1, E-selectin, and monocyte chemoattractant protein (MCP)] and monocyte chemotactic peptides (Berezova and Korovkin, 1998); (iii) attenuating neutrophil and monocyte adhesion, infiltration, and aggregation preventing conversion of the latter into the macrophages; (iv) suppressing platelet aggregation and adhesion; and (v) decreasing the expression of platelet-activating factor and thrombus formation. Inhibition of NOS in healthy volunteers significantly increases blood clotting time and worsens other coagulogram indices (Malakhov et al., 2009).

The ability of NO to directly interact with iron mediates many physiological and toxic effects. NO reversibly binds to heme iron of guanylate cyclase, cyclooxygenase, catalase, lipoxygenases, NOSs, cytochrome P450, peroxidases, and cytochromes of mitochondrial respiratory electron transport chain as well as to heme iron of oxyhemoglobin. Among these enzymes, guanylate cyclase is of exceptional importance. Due to binding of NO to Fe^2+^ of the guanylate cyclase porphyrin ring, the activity of the enzyme increases by a factor of hundreds. As a result, synthesized cyclic guanosine monophosphate regulates the vascular tone, immune reactions, neuronal mediation, platelet aggregation, platelet-endothelial interactions, and functioning of the diverse muscle cell types between other processes. Unlike guanylate cyclase, inhibition of all other heme-containing enzymes does not depend on heme iron valence in their active site when they interact with NO (Malakhov et al., 2009).

Studies showed that NO enhance cGMP synthesis through the activation of soluble guanylate cyclase (Randriamboavonjy and Fleming, 2005; Gkaliagkousi et al., 2007). Further, cGMP can downregulate cGMP-dependent Ca^2+^ channels or activate protein kinases G (cGMP-dependent protein kinases) (Bode-Böger et al., 1998; Buvaltsev, 2011). In human platelets, there is evidence of NO/cGMP-dependent inhibition of phospholipase C and, possibly, inositol trisphosphate receptor (InsP3R) phosphorylation resulting in the receptor desensitization. Additionally, protein kinases G upregulate protein phosphatase activating K^+^-channels by dephosphorylation. All these processes eventually decrease the cytoplasmic levels of free Ca^2+^ (Malakhov et al., 2009).

The activating effects of NO on cADP-ribosyltransferase through the cGMP-dependent mechanisms were also demonstrated. Synthesis of cADP-ribose, an agonist of ryanodine receptors, augments Ca^2+^ release from endoplasmic reticulum. Therefore, NO can both decrease and augment free Ca^2+^ concentration in cytosol through the cGMP-dependent pathway (Granik et al., 1997). One of the explanations of this phenomenon is organospecificity, related, in particularly, to diverse development of the endoplasmic reticulum and plasma membrane as well as peculiarities of the intracellular metabolism in different cell types. One can assume that the sophisticated and multidirectional effects of NO provide spatial and temporal regulatory tuning of the cytosolic Ca^2+^ levels ultimate for proper cell functioning (Malakhov et al., 2009).

There is evidence of decrease in the insulin-stimulated NO production by the endothelial and smooth muscle cells in the presence of insulin resistance (Bondarenko et al., 2002). In diabetes, platelet adhesion and spontaneous aggregation increase as well. Altered activity of NOS in platelets from patients with MetS can play the key role in onset of the platelet hyperactivation and development of macro- and micro-angiopathies. Studies of NO production in the platelets showed decreased basal NO production in all groups of MetS patients compared with healthy donors. Moreover, the lowest rates of NO synthesis were observed in patients with decompensated T2DM (Ogurkova et al., 2007). There is reverse correlation between the NO-mediated synthesis of cGMP in platelets and the levels of glucose and glycated hemoglobin in blood (Gkaliagkousi et al., 2007; Naseem and Riba, 2008). Reduced basal formation of NO by the cells from MetS patients can be caused by various reasons. The presence of hyperglycemia due to glucose autoxidation contributes to the formation of superoxide anion interacting with NO and mediating formation of the peroxinitrite attenuating NO content (Schaeffer et al., 1999). There is evidence that platelets express functional Ca^2+^-calmodulin-dependent constitutive NOS. Glycosylation of calmodulin can cause reduction of basal NO secretion. Additionally, the metabolic abnormalities can affect NOS expression in megakaryocytes and downregulate activity of the enzyme in platelets (Granik et al., 1997; Ogurkova et al., 2007). The experimental and clinical studies suggest that prolonged hyperglycemia contributes to the activation of the polyol pathway of glucose metabolism with depletion of NADP which is an obligatory cofactor of eNOS (Bondarenko et al., 2002). *In vitro* and *in vivo* studies demonstrated that oxidized low-density lipoproteins suppress platelet NO synthesis and stimulate platelet aggregation and formation of thromboxane A2 and serotonin (Malakhov et al., 2009).

Human platelet aggregation is also modulated by NO and prostacyclin (PGI2) released from the endothelium. Endothelial dysfunction, accompanying the insulin resistance syndrome, attenuates NO and PGI2 production in the endotheliocytes (Dandona and Aljada, 2004). Effects of NO and PGI2 on the platelets are mediated by the adenylate and guanylate cycases that synthesize cAMP and cGMP. The adenylate and guanylate cyclases are regulated by prostacyclin and NO, respectively. Formed cGMP inhibits phosphodiesterases metabolizing cAMP (Naseem and Riba, 2008; Stalker et al., 2012). Cyclic nucleotides stimulate cAMP-dependent PKA and cGMP-dependent protein kinase G (PKG) that, in turn, phosphorylate broad range of proteins. Phosphorylation results in an inactivation of small G proteins that belong to Ras and Rho families, inhibition of Ca^2+^ release from the intracellular stores, and modulation of actin cytoskeleton (Smolenski, 2012). Degradation of cAMP and cGMP is mediated by phosphodiesterases (Smolenski, 2012). Abnormalities in cAMP/cGMP pathways can cause platelet hyperactivity (Dandona and Aljada, 2004; Inada et al., 2008; Stalker et al., 2012; Shaturny et al., 2014). For example, De La Cruz et al. observed a decrease in cGMP content in patients with diabetic retinopathy and decrease in activity of platelet adenylate cyclase (De La Cruz et al., 2002). According to literature, platelets from patients with diabetes have decreased sensitivity to NO and PGI2. In diabetes mellitus, the number of PGI2 receptors is not decreased suggesting that the defect is downstream of the receptor. Livingstone et al. detected a decrease in G_*i*_ protein in the platelet membranes from T2DM patients. Some studies demonstrated elevated activity of cGMP-dependent phosphodiesterase eventually leading to decrease in sensitivity to NO (Vinik and Erbas, 2013). State-of-the-art proteomic approaches with the use of antibodies against eNOS suggest the presence of 135-kDa protein in human platelets though data are inconclusive on whether the protein is eNOS (Naseem and Riba, 2008). Inhibitors of NOS do not affect human platelet activation at all and there are no differences between the agonist-induced platelet aggregation in wild type and NOS-deficient mice. It is important to note that iNOS and eNOS mRNAs are found in human platelets by the standard methods (Gkaliagkousi et al., 2007) though the use of new technologies does not confirm this fact. The hypothesis, based on this fact, states that some platelet agonists directly affect soluble guanylate cyclases triggering NO-independent activation of cGMP-dependent signaling pathway (Naseem and Riba, 2008). Though NOS regulation in platelets is currently under discussion, the insulin-induced cGMP synthesis is decreased and agonist-induced platelet aggregation is insensitive to NOS inhibitors in T2DM patients (Randriamboavonjy and Fleming, 2009).

In our study, N-monomethyl-L-arginine (L-NMMA), a blocker of eNOS and iNOS, was used for evaluation of the involvement of NOS into antiplatelet effects of insulin. Both in healthy volunteers and patients with MetS, the collagen-induced platelet aggregation did not change in response to insulin. These data agree with the hypothesis suggesting absence of NOS in platelets (Kawamato et al., 2002). Similarly to its macrophage form, iNOS was found on the mitochondrial inner membrane (Joannides et al., 1995). Platelets contain significant amount of mitochondria and, therefore, iNOS can be present in these organelles. So what is the reason that the enzyme is difficult to detect in platelets? As known, platelet lifetime is not long and lasts for no more than 7–10 days. Half-life time of NOS is quite short (15–20 h); the enzyme is subject to the phosphorylation on tyrosine, serine, or threonine residue and can be easily oxidized by nitric monoxide produced by NOS itself or by other oxidants (anion-radical, hydrogen peroxide) (Malakhov et al., 2009). *De novo* protein synthesis in platelets is significantly limited due to the absence of nucleus. It is possible, that NOSs are present in younger platelets, but, due to rapid degradation of the enzymes and small lifetime of the platelets, NOSs are impossible to detect.

Hyperhomocysteinemia is often associated with T2DM and represents a risk factor for cardiovascular catastrophes (Boushey et al., 1995). Homocysteine affects blood vessels through the active oxygen forms, decrease in endothelial NO production, and augmentation of smooth muscle cell proliferation (Welch and Loscalzo, 1998). The study of B. Mutus et al. showed an association of *in vivo* and *in vitro* decreases in the NO production with the elevated homocysteine levels in healthy volunteers and diabetic patients (Mutus et al., 2001). At the same time, NO production after incubation with homocysteine is lower in cells from diabetic patients than in healthy individuals. Homocysteine can decrease platelet NO production by several mechanisms: (i) direct binding with formation of S-nitrohomocysteine; (ii) formation of superoxide anion and hydrogen peroxide followed by decrease in bioavailability of NO (Paolocci et al., 2001); and (iii) activation of iNOS accompanied by the synthesis of superoxide anion and NO whose interaction results in formation of peroxinitrate (Schaeffer et al., 1999; De La Cruz et al., 2002). Data demonstrated that homocysteine causes atherogenic effects in diabetic patients due to decreased platelet NO production with following augmentation of platelet activity and aggregation (Mutus et al., 2001).

Human platelets express insulin receptors on the cell surface, suggesting possible involvement of insulin in the platelet regulation. According to existing experimental evidence of insulin effects on platelet aggregation, in case of incubation with physiologic concentrations of insulin for 3–20 min, platelets respond with decreased aggregation induced by the following agonists: ADP, adrenalin, and collagen. Insulin also decreases thrombin- and angiotensin II-induced platelet aggregation (Udvardy et al., 1985; Trovati et al., 1998; Shitikova, 2000). Data show that anti-aggregation effects of insulin can be mediated through different intracellular mechanisms. In smooth muscle cells, insulin increases cGMP and cAMP contents by NO-dependent mechanism activating NOS and transport of L-arginine into the cells (Scherrer and Sartori, 1997; Westerbacka et al., 2002). The effects of insulin on platelets in healthy individuals consist in the NO-mediated elevation of cGMP and cAMP, decrease in Ca^2+^ currents induced by Ca^2+^ mobilizing agents, decrease in agonist-induced aggregation, augmentation of platelet binding with anti-aggregation prostanoids, and suppression of platelet binding with catecholamines leading to adrenalin-induced platelet aggregation (Udvardy et al., 1985; Shitikova, 2000; Suslova et al., 2007). In insulin resistant patients, calcium content in platelets rises in response to stimulation by insulin, leading thereby to platelet activation and aggregation (Schaeffer et al., 1999; Westerbacka et al., 2002; Paneni et al., 2013).

Decrease in the number and sensitivity of insulin receptors on platelets in T2DM patients can result in platelet hyperactivation (Udvardy et al., 1985). On the other hand, a decrease in platelet sensitivity to insulin can be caused by abnormal intracellular insulin signaling by second messenger system (Bode-Böger et al., 1998; Trovati et al., 1998; Dandona and Aljada, 2004; Ogurkova et al., 2012). The study of individuals with abnormal carbohydrate metabolism demonstrated augmentation of platelet activity and aggregation in response to an increase in content of cGMP exerting anti-aggregation effect. The authors assume that elevated platelet aggregation in these patients causes cGMP increase by the negative feedback mechanism (Anwaar et al., 2000). Westerbacka J. et al. studied the regulation of platelets in individuals with and without obesity. They showed that the *in vivo* infusion of insulin suppresses platelet adhesion on collagen in individuals with normal weight, but not in obese patients. Moreover, only in the absence of obesity, insulin significantly elevates cGMP concentration in platelets contributing to platelet inactivation following the adhesion. These data suggest the hypothesis of insulin-mediated suppression of the interaction between platelets and collagen in normal conditions. In obesity, this effect of insulin is absent suggesting the existence of additional mechanism of pathogenic interplay between insulin resistance and atherothrombosis (Anfossi et al., 1998; Westerbacka et al., 2002). According to our data on healthy volunteers and patients with MetS and heart failure (Ogurkova et al., 2012), platelets, incubated with the physiological concentrations of insulin (1 nM) for 3 min, respond with decrease in a degree and rate of collagen-induced platelet aggregation, determined based on the curves of the mean aggregate size and light transmission. Also, literature presents reports describing a decrease in platelet aggregation induced by other activators (ADP, thrombin, arachidonate, and platelet-activating factor) in the presence of insulin (Vinik and Erbas, 2013).

In our study, isolated platelets were incubated with insulin and treated with blockers of cGMP- and cAMP-dependent phosphodiesterases. Phosphodiesterases inhibitors increased intracellular concentrations of cyclic nucleotides and significantly decreased collagen-induced platelet aggregation both in MetS patients and healthy volunteers (Ogurkova et al., 2012). A scheme illustrating the interactions of the signaling pathways and effects of the modulators is presented in Figure 1. More pronounced decrease in collagen-induced aggregation of platelets, incubated in the media containing mix of insulin and phosphodiesterase inhibitors, suggests a synergy of the inductors (Ogurkova et al., 2012). The overlapping observations on the insulin-induced platelet aggregation and cGMP contents in healthy individuals and patients with insulin resistance confirm that cGMP increase in platelets is the main mechanism of insulin-induced modulation of platelet function. M. Trovati et al. demonstrated similar effects of various insulin concentrations on platelet aggregation and cGMP contents in different subjects, namely: supraphysiological concentrations (1920 pmol/L) in obese or diabetic patients with obesity and more physiological concentrations (240–480 pmol/L) in healthy individuals (Trovati et al., 1995).

**Figure 1:**
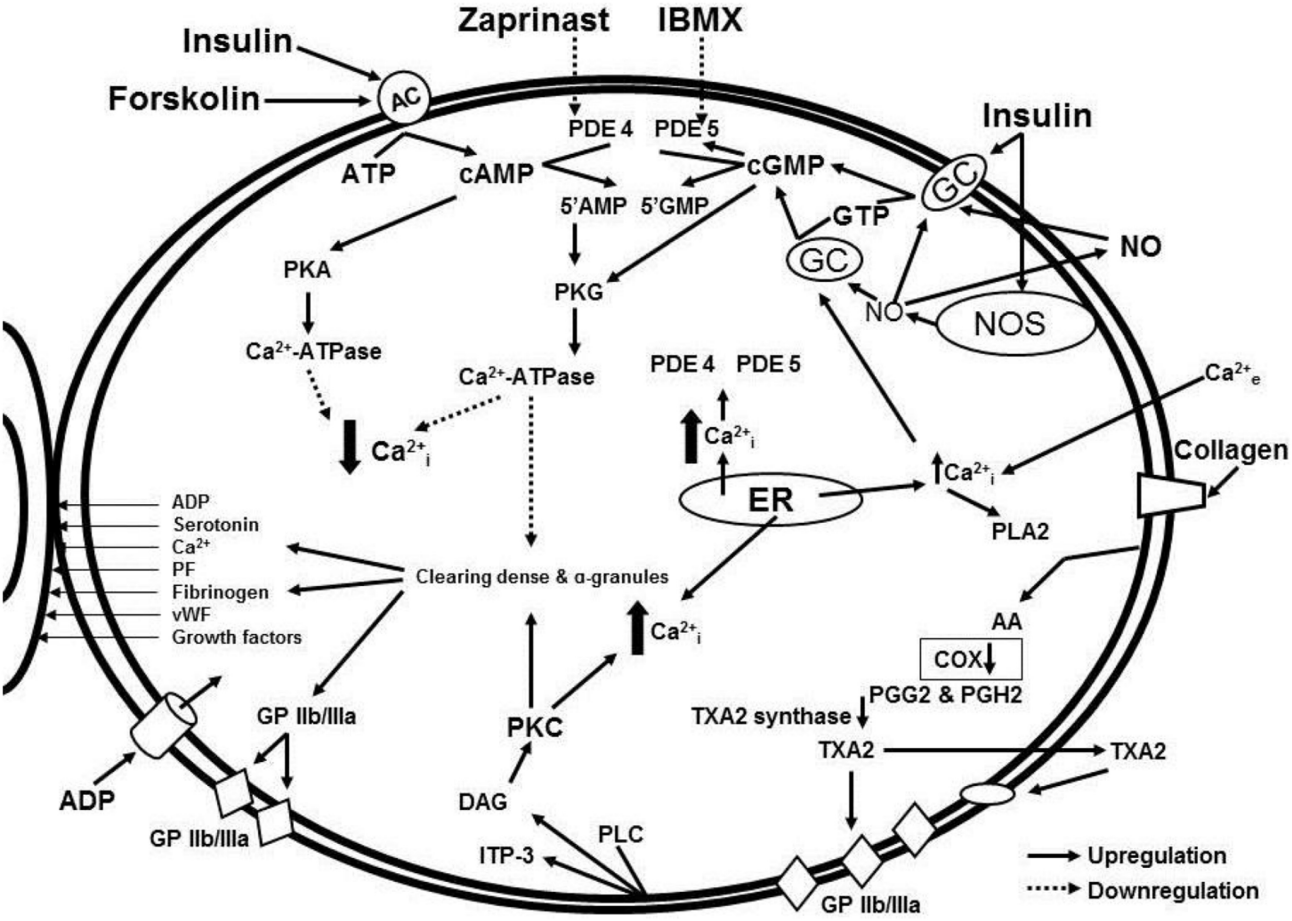
**Signaling pathway interaction and effects of modulators:** cGMP and cAMP-dependent intracellular signaling systems in the insulin-mediated regulation of platelet aggregation in patients with metabolic disorders.

Some authors consider platelets as the source of active synthesis of humoral factors simultaneously stimulating the processes of thrombus formation and inflammation (Soslau et al., 1997). Recently, platelet ability to release anti-inflammatory cytokine interleukin-1 (IL-1) and other inflammatory mediators was discovered. Platelets also express adhesive molecules, chemokines, and growth factors (Anfossi et al., 2010; Beaulieu et al., 2014). According to the study by Lea M. Beaulieu et al. IL-1β augments agonist-induced platelet aggregation by 1.2 times in response to collagen and by 4.2 times in response to thrombin. However, different concentrations of IL-1β do not affect aggregation of the isolated platelets. These data lay the foundation for the hypothesis that IL-1β modulates effects of aggregation agonists by influencing the signaling cascades (Beaulieu et al., 2014). Interleukin-6 (IL-6) can stimulate thrombocytopoesis; in the presence of IL-6, nascent platelets have an increased sensitivity to thrombin and procoagulant activity (Esmon, 1999, 2004; Anfossi et al., 2010). It is known that proinflammatory cytokines participate in the development of dyslipidemia and insulin resistance and are the markers of T2DM and its complications. Studies of anti-inflammatory cytokine tumor necrosis factor α (TNF-α) demonstrated its relationships with obesity and insulin resistance. Adipose tissue produces large amounts of TNF-α whereas knockout mice deficient on TNF-α or its receptor are protected from developing the insulin resistance (Nieto-Vazquez et al., 2008; Anfossi et al., 2010). According to various studies, this adipocytokine stimulates biosynthesis of plasminogen activator inhibitor-1 (PAI-1) in different cell cultures and in many mouse tissues *in vivo* as well as blocks insulin effects by decreasing activity of insulin receptor tyrosine kinase. The relationships between MetS and fibrinolysis deficiency, caused by the elevated level of PAI-1, are currently known. Plasminogen activator inhibitor-1 is the main physiologic plasminogen activator inhibitor *in vivo* suppressing fibrin and contributing to fibrin fibers dissolving (Esmon, 2004; Petrik and Pavlishchuk, 2008; Paneni et al., 2013). The studies *in vitro* showed that TNF-α stimulates platelet aggregation and production of active oxygen forms mostly through the activation of arachidonic acid metabolism (Pignatelli et al., 2005; Pircher et al., 2012). On the other hand, according to data of Cambien B. et al. generated in the experiments of mice, tumor necrosis factor in high concentrations (1 ng/mL) exerts the anti-thrombotic action, decreases platelet aggregation activity, and inhibits thrombus formation. The authors assume that anti-thrombotic action of TNF-α is mediated indirectly through the rapid NO release by the endothelial cells of the vascular wall rather than through the interaction with platelets (Cambien et al., 2003). Busse and Mülsch demonstrated the evidence of cytokine (TNF-α, IL-1)-induced expression of eNOS in aortic endothelial cells (Busse and Mülsch, 1990). We studied the effects of TNF-α and IL-1β on the aggregation activity of isolated platelets from patients with MetS and healthy volunteers. According to our data (Periodic Report 2 - SICA-HF ^1^), incubation of isolated platelets with TNF-α in concentrations of 0.05 and 1 ng/mL in the reaction media decreases collagen-induced cell aggregation in group of patients with metabolic abnormalities and in healthy volunteers, though this response is significantly more pronounced in MetS patients. Additional application of NOS inhibitor, L-NMMA, in the incubation medium affected collagen-induced platelet aggregation in the presence of TNF-α and IL-1β neither in group of patients with MetS nor in control group. According to our data (Periodic Report 2 - SICA-HF), TNF-α and IL-1β exert NO-independent anti-aggregation effects on isolated human platelets independently on the presence of insulin resistance in the subjects.

Gareth J. Padfield et al. studied the effects of intravenous infusion of etanercept (TNF-α receptor antagonist) in patients with acute myocardial infarction. Infusion of etanercept in a dose of 10 mg decreased the neutrophil count and IL-6 level, but enhanced the platelet-monocytic aggregation (Padfield et al., 2013). Joachim Pircher et al. studied the effects of TNF-α (5 ng/mL) on hemostasis in mice and concluded that this cytokine exerts prothrombotic action. The authors explained prothrombotic action of TNF-α by endothelial activation. However, their results were still inconclusive in regard whether the effects on the platelets were direct (Pircher et al., 2012). Pasquale Pignatelli et al. studied platelet activation in patients with heart failure in response to stimulation by tumor necrosis factor. Researchers found that physiologic concentrations of TNF-α (20–40 pg/mL) do not affect platelet aggregation (Pignatelli et al., 2005). Therefore, the currently available data cannot unambiguously resolve the question whether the effects of TNF-α are prothrombotic or antithrombotic (Anfossi et al., 2010). This question remains to be explored.

## Conclusion

Platelet activation causes high risk of cardiovascular complications in MetS. T2DM is associated with the changes in the intracellular signaling systems regulating platelet functions. Due to the altered NOS expression and activity, platelets increase their prothrombogenic potential. The cGMP-mediated antiaggregational effects of insulin, involving NOS and NO-dependent mechanisms, become abnormal. The effects of aggregation-triggering agonists are modulated by the proinflammatory factors. All these mechanisms of changes in platelets aggregation activity in MetS and DMT2 are caused by the metabolic disturbances including insulin resistance, hyperglycemia, and dyslipidemia. Future studies of the second messenger systems involved in the platelet aggregation/anti-aggregation mechanisms are required. Further investigations should be focused on an identification of the abnormalities in these systems at early stages of metabolic disorders in patients. Results of such studies may lay a foundation for a discovery of new approaches for prevention and treatment of cardiovascular complications in MetS and DMT2.

### Conflict of interest statement

The authors declare that the research was conducted in the absence of any commercial or financial relationships that could be construed as a potential conflict of interest.

## Footnotes

^1^Periodic Report 2 - SICA-HF (Studies investigating co-morbidities aggravating heart failure). Record Number: 94060. Last updated on: 2013-11-28. Available at http://cordis.europa.eu/publication/rcn/14456_en.html.

## Abbreviations

5′AMP: adenosine 5′ monophosphate
5′GMP: guanosine 5′-monophosphate
AC: adenylate cyclase
ADP: adenosine diphosphate
ATP: adenosine triphosphate
Ca^2+^-ATPase: calcium ATPase
cAMP: cyclic adenosine monophosphate
cGMP: cyclic guanosine monophosphate
COX: cyclooxygenase
DAG: diacylglycerol
ER: endoplasmic reticulum
GC: guanylate cyclase
GP Iib/IIIa: glycoprotein IIb/IIIa (also known as integrin αIIbβ3)
GTP: guanosine triphosphate
ITP-3: inositol trisphosphate
NO: nitric oxide
NOS: nitric oxide synthase
PDE4: phosphodiesterase 4
PDE5: phosphodiesterase 5
PF: platelet factor
PGG2: prostaglandin G2
PGH2: prostaglandin H2
PKA: protein kinase A
PKG: protein kinase G
PLA2: phospholipase A2
PLC: phospholipase C
TXA2: thromboxane A2
vWF: von Willebrand factor.
